# The Nordic Subpopulation Research Programme: prediction of treatment outcome in patients with low back pain treated by chiropractors - does the psychological profile matter?

**DOI:** 10.1186/1746-1340-17-14

**Published:** 2009-12-30

**Authors:** Charlotte Leboeuf-Yde, Annika Rosenbaum, Iben Axén, Peter W Lövgren, Kristian Jørgensen, Laszlo Halasz, Andreas Eklund, Niels Wedderkopp

**Affiliations:** 1 The Department of Research, the Spine Center, Hospital Lillebælt, University of Southern Denmark, Denmark; 2 Private practice, Linköping, Sweden; 3 The Karolinska Institutet, Stockholm, Sweden; 4 Private practice, Stockholm, Sweden; 5 Private practice, Lund, Sweden; 6 Private practice, Södertälje, Sweden

## Abstract

**Background:**

It is clinically important to be able to select patients suitable for treatment and to be able to predict with some certainty the outcome for patients treated for low back pain (LBP). It is not known to what degree outcome among chiropractic patients is affected by psychological factors.

**Objectives:**

To investigate if some demographic, psychological, and clinical variables can predict outcome with chiropractic care in patients with LBP.

**Methods:**

A prospective multi-center practice-based study was carried out, in which demographic, clinical and psychological information was collected at base-line. Outcome was established at the 4^th ^visit and after three months. The predictive value was studied for all base-line variables, individually and in a multivariable analysis.

**Results:**

In all, 55 of 99 invited chiropractors collected information on 731 patients. At the 4^th ^visit data were available on 626 patients and on 464 patients after 3 months. Fee subsidization (OR 3.2; 95% CI 1.9-5.5), total duration of pain in the past year (OR 1.5; 95% CI 1.0-2.2), and general health (OR 1.2; 95% CI 1.1-1.4) remained in the final model as predictors of treatment outcome at the 4^th ^visit. The sensitivity was low (12%), whereas the specificity was high (97%). At the three months follow-up, duration of pain in the past year (OR 2.1; 95% CI 1.4-3.1), and pain in other parts of the spine in the past year (OR1.6; 1.1-2.5) were independently associated with outcome. However, both the sensitivity and specificity were relatively low (60% and 50%). The addition of the psychological variables did not improve the models and none of the psychological variables remained significant in the final analyses. There was a positive gradient in relation to the number of positive predictor variables and outcome, both at the 4^th ^visit and after 3 months.

**Conclusion:**

Psychological factors were not found to be relevant in the prediction of treatment outcome in Swedish chiropractic patients with LBP.

## Background

It is our experience that many chiropractors have a biomechanical rationale for their treatment of low back pain (LBP). For most chiropractors therefore, the purpose of spinal manipulation would primarily be to normalize the mechanics of the spine. Massage or other adjunct therapies are often used to enhance or facilitate the effect, and patients' own activities would be seen as an integral part of the therapy. Typically, during the initial stage, manipulation is used to reduce the pain and increase the patients' ability to move about in a more relaxed manner, whereupon they are encouraged to combine rest with movement. As the pain subsides, patients are encouraged to increase their activities in an individually tailored fashion, to avoid the problems to recur. Sometimes specific movements or exercises are prescribed to counteract unsuitable postures or weaknesses of the spine.

This biomechanical approach is governed by a wish to obtain optimal function of the spinal structures with respect for any underlying anatomical or patho-anatomical limitations. Therefore, non-recovery or recurring symptoms would often be seen as the result of unsuitable activities, movements or prolonged postures, or the inevitable return of spinal dysfunction because of spinal degeneration.

However, this concept is hardly supported by the present day concepts, according to which non-recovery of LBP is considered also or, perhaps even mainly, a psychosocial problem [1]. For example, contrary to shoulder pain, the poor outcome of LBP was in one study shown to be associated with psychological factors [2]. Therefore, this predominantly biomechanical approach may be insufficient and in dire need to be investigated also for chiropractic patients.

A number of outcome studies of patients treated by chiropractors for LBP have been performed in the Nordic countries, in which some predictors of outcome were identified. In all these studies, field practitioners participated in selecting the potential predictors, based on their own experience and opinion. In relation to persistent LBP, some non-spinal factors were identified as predictors of poor outcome (the female gender and the use of social welfare services) [3]. Further, past history (longer duration of pain and of disability) [3], present symptoms (pain in the leg) [4,5], and body type (overweight/obesity) [5] have been shown to be associated with outcome in chiropractic patients treated for LBP. The single most dominant factor isolated, so far, in these studies was absence of immediate improvement [5].

In relation to psychological factors, we were able to find only one study on the predictive value of psychosocial factors in chiropractic patients with LBP [6]. According to this study, with data from one British clinic on 101 patients at 6 weeks follow-up, fear-avoidance and anxiety were not an issue because these traits were absent in the study subjects. It would be necessary, therefore, to include a larger study sample from a wide variety of clinics, and to study a larger range of psychological factors in order to establish if certain psychological profiles can predict outcome.

For this reason, two questionnaires were selected in order to describe the psychological profile of patients seeking care for LBP with Swedish chiropractors (to be reported elsewhere). A second purpose was to investigate if some demographic, psychological, and clinical variables can predict outcome with chiropractic care in patients with LBP.

## Methods

### Participating chiropractors

A project group consisting of six practising chiropractors and two senior researchers designed and carried out the study. One of the chiropractors was responsible for the logistics of the study. These practising chiropractors were responsible for the supervision of the data collection, using a previously described method [7,8]. In this study, previously compliant chiropractors throughout Sweden collected the data for the study.

### Participating patients

Consecutive patients who sought care for LBP with or without pain radiating into the leg, who were new to the clinic or had not received chiropractic treatment within the past three months, were invited to participate in the study in the spring of 2006, providing that they returned for treatment at least once.

### Data collection and ethical considerations

The data collecting chiropractors were provided with a written instruction and a set of questionnaires. They were also followed with regular telephone calls to ensure that data collection proceeded smoothly.

The base-line questionnaire and the questionnaire at the 4^th ^visit were completed by the chiropractor whilst interviewing the patient and returned to the research officer. A psychological questionnaire was completed by the patients whilst in the clinic. This was put in an envelope and sealed by the patient, handed in to the chiropractor or receptionist to be mailed to the research team together with the other information.

If patients did not receive 4 treatments, the 4^th ^visit questionnaire was completed when treatment was completed.

After 3 months, a brief anonymous follow-up questionnaire was mailed to the patients, and another one at 12 months (data not reported here). Therefore, no reminders were sent out to the non-responders. After the 12 months' follow-up, the names and addresses of patients were destroyed, and all data were analyzed and reported anonymously. All participating patients signed an informed consent. Approval of the study was given by the regional ethics committee in Linköping, Sweden (M57-06).

### Variables of interest

Predictor variables were collected at base-line in relation to outcome at the 4^th ^visit. The same base-line variables were used as predictor variables in relation to outcome at 3 months with the addition of two variables obtained at the 4^th ^visit (pain intensity past 24 hrs at 4^th ^visit and outcome at 4^th ^visit). The outcome variable at the 4^th ^visit was obtained from the question "Improvement? (compared to the 1^st ^visit): Definitely better/probably better/unchanged/probably worse/definitely worse/missing answer". At three months, the outcome variable was obtained from the question:"Generally, how has your low back been recently? Definitely worse/probably worse/unchanged/probably better/definitely better".

### Questionnaires

Data were collected at three points in time, at the first visit, the fourth visit and after 3 months.

A. The base-line questionnaire consisted of three parts, namely:

1. Demographic and life-style information (gender, age, fee subsidization, type of work, and tobacco consumption).

2. Clinical information at the first visit (leg pain, pain intensity past 24 hrs, duration of pain for the current episode, duration of pain in total past year, problems in neck/mid back past year, general health, and type of treatment at first visit).

3. Two questionnaires on the psychological profile: The Hospital Anxiety and Depression Scale (HADS) used to measure state anxiety and depression, and PASS-20 used to measure 1. cognitive anxiety, 2. escape avoidance, 3. fearful thoughts, and 4. physiological symptoms and signs of pain.

The HADS, developed by Zigmond and Snaith in 1983 [9], contains a seven-item depression subscale and a seven-item anxiety subscale, with each question having four response possibilities going from, for example "not at all" or "very rarely" to an affirmative response, such as "most of the time", "very often", or "absolutely". These are graded from 0 to 3, resulting in a maximum possible score of 21 for each scale. Cut-off points have been established for these two scales: <6 normal, 6-10 borderline, and >10 probable anxiety or depression diagnosis [10].

The four dimensions measured in the PASS-20 questionnaire each consists of five questions, graded from 0 ("never") to 5 ("always"), with a maximum of 20 points per dimension. No cut-off points for clinical relevance appear to have been published for this questionnaire [10].

B. The first follow-up at the 4^th ^visit consisted of clinical information (duration since first treatment, pain intensity past 24 hrs, present LBP status, number of treatments, and if treatment was completed before the 4^th ^visit, the reason for this, and self-reported outcome status as compared to the first visit).

C. The second follow-up questionnaire at 3 months consisted of a pre-stamped postcard with a short introductory text: "In the month of ........., you consulted a chiropractor for LBP. You then gave written permission for us to contact you for research purposes. The research group would now like to have some answers to a couple of questions relating to your problems." Two questions followed, one on pain intensity past week and one on the low back pain status in general (described above). The first question was considered merely a "memory jogger" in preparation for the second question, which was the outcome question at 3 months.

### Rationale for choice of variables

Of the predictor variables, the demographic and clinical variables were originally selected by the chiropractors who participated in previous research groups because they expected these variables to influence the outcome of patients with LBP, whereas the psychological questions were taken from questionnaires concurrently used by other research teams at the University of Linköping, Sweden. Another three variables, not previously tested in our series of studies, were included because the present research team suspected that they could influence outcome. These were fee subsidization (financial contribution was thought to encourage compliance and positive attitudes to the treatment), type of work (heavy work might make recovery difficult), and use of tobacco (because it may be a proxy for an unhealthy life-style in general and because reduced oxygenation of muscles was thought capable of slowing down the healing process).

In order to be able to compare our results to those of our previous studies, the outcome question used at the 4^th ^visit is the same as that used in a number of previous studies of this type [4,5,11,12], and the outcome question used at 3 months was designed to be equally brief, to enhance the response rate.

### Validity of data

The demographic and clinical information questions were mainly based on previously used questions. These and the outcome question at the 4^th ^visit have been validated through their repeated use with logical and similar results [4,5,11,12]. The validity of the HADS questionnaire has been shown previously to be acceptable [13] as well as that of the PASS-20 questionnaire [14].

### Analysis and presentation of data

Data were analyzed with Stata version 11. All variables were described and some of the categorical variables collapsed for further analyses. These transformations can be seen in the relevant tables of results. The HADS anxiety and depression indices were reported according to the predefined values (normal, borderline, and clinically significant anxiety or depression). The other four psychological variables from PASS-20 were transformed into percentages to be analyzed as continuous data.

A comparison was made for the base-line variables between 1) those who participated in the study both at base-line and at 3 months and 2) those who participated only at base-line (drop-outs).

Outcome at the 4^th ^visit was dichotomized into "good outcome" (definitely better) and "not good outcome" (all other 4 options including missing answer). Outcome at 3 months was defined similarly but did not include missing answers. Cross-tabulation analyses were made to investigate predictors of (poor) treatment outcome at the 4^th ^visit and at 3 months.

Associations with p-values of p = 0.05 or smaller were entered in a multivariate logistic regression and, finally, a stepwise regression was performed, in which all possible combinations of variables were tested.

First, the sociodemographic variables and the clinical variables were tested, using odds ratios, sensitivity, specificity, and the area under the receiver operating characteristics (ROC) curve as measures of prediction. Thereafter, the psychological variables were added to the model, to see if this would improve the predictive value. Anxiety and depression were dichotomized into clinically significant anxiety/depression (yes/no). All multivariable analyses were adjusted for the possible cluster effect that could arise because of the effect that single clinicians might have on the results, using the robust standard errors as cluster options as described in the STATA manual [15].

The performance of the model was further investigated by studying the (good) outcome at the 4^th ^visit and at 3 months in relation to the number of statistically significant risk factors that were present in each individual. For ease of interpretation this was reported as percentages in a frequency table. A brief explanation of some of the methodological terms has been provided in Additional file 1, whereas more detailed explanations can be found in standard text-books.

A description of the base-line psychological variables and their interactions with the clinical base-line variables will be presented elsewhere. A third study, dealing with predictors of prolonged (maintenance) care will also be published.

## Results

### Descriptive data

Out of the 99 invited chiropractors, 55 participated in the study, providing valid questionnaires for 731 patients at base-line. Data were available on 626 patients at the 4^th ^visit and 464 returned the follow-up questionnaire after 3 months.

At base-line, 1/3 of the patients had pain radiating into the leg. The majority had moderate or severe pain. Almost 50% reported the duration of pain for the present problem to have lasted at least 2 weeks and the total duration in the past year was at least 30 days for 50%. Fifty percent reported to have had some problems past year also in the neck and/or mid back. Seventy-eight percent said that their general health was very good or good. For a detailed description of their base-line status and the type of treatment given, see Tables 1 and 2.

**Table 1:** Description of the 731 chiropractic patients who took part in a practice-based outcome study on low back pain

Variables	Number of respondents	Number	Percentage
Gender	727		
men		374	51
women		353	49

Age	621		
11-20 yrs		11	2
21-30 yrs		83	13
31-40 yrs		156	25
41-50 yrs		133	21
51-60 yrs		113	18
61-70 yrs		90	14
71-80 yrs		30	5
81-90 yrs		5	1

Fee subsidization	713		
total		58	8
partial		164	23
none		491	69

Main work	704		
physically hard		69	10
mixed hard/less hard		213	30
walking/standing		166	24
mainly sitting		256	36

Smoking	706		
never smoked		403	57
ex-smoker		198	28
sometime smoker		26	4
smoke up to 20/day		72	10
smoke > 20/day		7	1

Smokeless tobacco	711		
yes		106	15
no		605	85

Demographic data.

**Table 2:** Description of the 731 chiropractic patients who took part in a practice-based outcome study on low back pain

Variables	Number of respondents	Number	Percentage
LBP	731		
yes		718	98
no (only leg pain)		13	2

Leg pain	731		
yes		271	37
no		460	63

Pain intensity past 24 hrs	727		
none		16	2
weak		86	12
moderate		303	42
severe		276	38
unbearable		46	6

Duration of pain this period	730		
1-7 days		262	36
8-14 days		108	15
more than 14 days		360	49

Duration of pain in total past 12 months	730		
maximum 30 days		367	50
more than 30 days		363	50

Problems past yr also in neck/mid back	728		
no		366	50
yes in total maximum 30 days		161	22
yes in total more than 30 days		201	28

General health	727		
very good		280	39
quite good		286	39
OK		116	16
quite bad		43	6
very bad		2	<1

Type of treatment at 1^st ^visit (several replies possible)	731		
SMT (incl. activator)		626	86
drop-piece table		69	79
soft tissue therapy		377	52
blocks/SOT		42	6

Clinical data at base-line

Only 9% and 4%, respectively, were classified as having clinically significant anxiety or depression. Also the results of the four psychological profiles from the PASS-20 questionnaire indicated that this was not a particularly afflicted population. For a description of the psychological profile, see Table 3.

**Table 3:** Description of the 731 chiropractic patients who took part in a practice-based outcome study on low back pain

Description of variables	Numbers of respondents	Minimum and maximum estimates obtained	Numbers in sub-groups	Mean/Median (95%CI)
Anxiety index (range 0-21)	722	0-19		Mean: 5.4 (5.2-5.7)
normal 0-7			404	
borderline 8-9			252	
clinical significance 10-21			66	

Depression index (range 0-21)	722	0-16		Mean:3.8 (3.5-4.0)
normal 0-7			541	
borderline 8-9			155	
clinical significance 10-21			26	

Cognitive anxiety (range 0-25)*	706	0-100*	NA	Mean 49.7 (48.1-51.2)

Escape avoidance (range 0-25)*	708	0-96*	NA	Mean: 46.7 (45.2-48.1)

Fearful thoughts (range 0-25)*	720	0-96*	NA	Median: *24 (22-26)*

Physiological symptoms and signs Of pain (range 0-25)*	714	0-96*	NA	Median: *24 (24-26)*

Psychological profile at base-line

*No known cut-off points for clinically significant threshold values, therefore these variables weree transformed into % and reported as mean or median values only. For this reason the range (reported as absolute values in column 1) and the estimated values (minimum, maximum, mean and median values reported in columns 3 and 5) do not seem to make sense.

The base-line questionnaires included information also about the 4^th ^visit. However, circa 1/3 of the patients received only 1-3 treatments. The most common reason for not returning was "not necessary, better" (n = 155; 56% of those who did not return for the 4^th ^visit). At this time, the pain intensity was reported to be weak or non-existing by 72%, and 76% reported to be "definitely better" (Table 4).

**Table 4:** Description of the 731 chiropractic patients who took part in a practice-based outcome study on low back pain

Variables	Number of respondents	Number	Percentage
Duration since first treatment (at 4th visit or before if treatment completed before then)	642		
1-14 days		402	63
15-28 days		176	27
4-6 weeks		47	7
6-8 weeks		9	1
more than 8 weeks		8	1

Pain intensity past 24 hrs (at 4^th ^visit)	626		
none		182	29
weak		267	43
moderate		148	24
severe		27	4
unbearable		2	<1

Present LBP status (outcome at 4^th ^visit)	662		
definitely better		506	76
probably better		107	16
no change		41	6
probably worse		5	1
definitely worse		3	<1

Number of treatments	730		
1		53	7
2		111	15
3		113	15
4		453	62

If treatment concluded before 4^th ^visit, why?	731		
not necessary, OK		155	21
not better		16	2
not possible to return/not able to pay		46	6
absent for unknown reason		48	7
referred out		13	2
not relevant (were not concluded before 4^th ^visit)		453	

Clinical data at the 4^th ^visit (or before, if treatment was concluded earlier)

The response rate at the 3 months follow-up was 63% (464 of the 731 from the base-line sample). The percentage reporting the pain intensity to be weak or non-existing was now 68, and 56% reported to be "definitely better". The status of the 267 drop-outs was unknown (Table 5).

**Table 5:** Description of the 464 chiropractic patients who took part in the 3 months follow-up survey of a practice-based outcome study on low back pain

Variables	Number of respondents	Number	Percentage
Pain intensity past week	464		
none		137	29
weak		181	39
moderate		118	25
severe		28	6
unbearable		0	0

Recent LBP status in general	464		
definitely better		258	56
probably better		106	23
unchanged		81	17
probably worse		16	3
definitely worse		3	1

### Comparison between respondents and drop-outs at 3 months

A comparison was made between the 464 patients, on whom data were available both from the base-line and 3 months follow-up, and the 267 persons, who were lost at the 3 months follow-up. The only differences were that drop-outs were more likely than respondents to be somewhat younger (mean age 42 yrs; 95% CI: 40-44 vs. 49 yrs; 48-51), men (60%; 55-66 vs. 40%; 34-45), and twice as likely to use smokeless tobacco (23%; 18-27 vs. 10%; 7-12).

There were no other differences between the groups in relation to type of work, smoking, fee subsidization, leg pain, pain intensity at base-line and at the 4^th ^visit, duration of pain at base-line, total duration of pain in the past year, pain in other parts of the spine past year, general health, and outcome at the 4^th ^visit. There were also no differences in relation to the psychological variables.

#### 1. Results at the 4th visit

##### i. Cross-tabulations between the potential predictors and outcome at the 4th visit

As can be seen in Table 6, one social factor (fee subsidization), and four health-related factors (leg pain, duration of LBP at base-line, total duration of pain in the past year, and general health) were individually associated with outcome at the 4^th ^visit. In addition, all the psychological variables but escape avoidance were significantly associated with outcome. Because all the psychological variables were found to be highly correlated (which could result in co-linearity problems), we decided to include only anxiety and depression in our continued analyses. They were also easier to work with as they had predetermined cut-points for abnormal findings.

**Table 6:** Cross-tabulations of base-line variables vs. good outcome at the 4^th ^visit for 731 chiropractic patients treated for low back pain

Variables	Subgroups	P-value
***Demographic background and life-style variables***		

Age	*Max. 40 yrs vs. older	0.1

Sex	*Men vs. women	0.1

Type of work	*Sitting vs. walking/standing vs. mixed hard/light work vs. hard physically hard work	0.6

Smoking	*No never vs. stopped/sometimes vs. daily	0.7

Smokeless tobacco	*No vs. yes	0.3

Treatment reimbursed	*Not or some reimbursement vs. total reimbursement	**0.000**

***Description of low back pain***		

Leg pain	*No vs. yes	**0.02**

Pain intensity past 24 hrs	*None/weak vs. moderate vs. severe/unbearable	1.0

Duration of pain at base-line	*1-7 days vs. 8-14 days vs. >14 days	**0.000**

Total duration of pain past year	*Max. 30 days vs. >30 days	**0.000**

Pain in other parts of spine past year	*No vs. yes max. 30 days vs. yes >30 days	0.09

General health	*Very good/good vs. OK vs. rather bad/very bad	**0.02**

***Psychological profile***		

Anxiety	**No vs. borderline vs. yes	**0.0007**

Depression	**No vs. borderline vs. yes	**0.0001**

Cognitive anxiety	***Continuous data	**0.02**

Escape avoidance	***Continuous data	1.0

Fearful thoughts	***Continuous data	**0.008**

Physiological symptoms and Signs of pain	***Continuous data	**0.005**

P-values smaller than 0.05 have been written in bold and the corresponding variables have been included in further multivariate analyses

* Chi-square test

**Logistic regression

*** Test for trend

##### ii. Multvariate analysis - 4^th ^*visit*

After the logistic regression of the non-psychological factors, three variables stayed in the model, namely: fee subsidization (OR 3.2; 95% CI 1.9-5.5), total duration of pain in the past year (OR 1.5; 95% CI 1.0-2.2) and general health (OR 1.2; 95% CI 1.1-1.4). The sensitivity was low (12%) but the specificity high (97%). The area under the ROC was 0.65, i.e. fairly low.

When the psychological variables anxiety and depression were added, the ORs remained low. The sensitivity, specificity and area under the ROC remained virtually unchanged (14%, 97% and 65%, respectively). Fee subsidization (OR 3.2; 2.1-5.1), total duration of pain in the past year (OR 1.8; 1.3-2.6), and anxiety (OR1.3;1.0-1.7) remained in the final model. A post hoc analysis with all the six psychological variables forced into the analysis did not change the results.

##### ii. Model performance - 4th visit

When the three variables from the first model (total fee subsidization, total duration of pain in the past year more than 30 days, and general health less than OK) were tested against outcome, a positive gradient was found. This went from 79% of patients without any of these attributes having a good outcome, to only 45% if they had 2 or 3 of these. If the variables anxiety or depression were added to the analysis, the results looked very similar, going from 82% of patients with good outcome if they had none of these findings to 36% if they had 4 or 5 of them (Table 7, Table 8 and Table 9).

**Table 7:** The percentage of patients with good outcome at the 4^th ^visit by the number of positive statistically significant predictor variables

Number of predictor variables present in patients	0	1	2 or 3
**Number of patients**	317	331	60

**Percentage of these patients with good outcome**	79	64	45

The predictor variables in this analysis were:**total subvention, total duration of pain in the past year, and general health**. A positive dose response was noted for all three combinations of predictor variables.

**Table 8:** The percentage of patients with good outcome at the 4^th ^visit by the number of positive statistically significant predictor variables

Number of predictor variables present in patients	0	1	2 or 3
**Number of patients**	292	307	69

**Percentage of these patients with good outcome**	79	69	35

The predictor variables in this analysis were: **total subvention, total duration of pain in the past year, and anxiety**. A positive dose response was noted for all three combinations of predictor variables.

**Table 9:** The percentage of patients with good outcome at the 4^th ^visit by the number of positive statistically significant predictor variables

Number of predictor variables present in patients	0	1	2	3	4 or 5
**Number of patients**	170	148	173	133	39

**Percentage of these patients with good outcome**	82	75	69	58	36

The predictor variables in this analysis were:total subvention, total duration of pain in the past year, general health, anxiety and depression. A positive dose response was noted for all three combinations of predictor variables.

#### 2. Results at 3 months

##### i. Cross-tabulations between the potential predictors and outcome at-3 months

The same analyses were repeated in relation to outcome at 3 months with an extra predictor variable being outcome at the 4^th ^visit (Table 10). This time there were positive associations with the outcome variable for the social factor (fee subsidization) and three of the health-related variables at base-line (duration of pain at base-line, total duration of pain in the past year, and general health), as well as for one previously non-significant variable: pain in other parts of spine in the past year. Outcome at the 4^th ^visit was also significantly associated with outcome at 3 months. However, none of the psychological variables was significantly associated with the outcome variable at this time.

**Table 10:** Cross-tabulations of potential predictors vs. good outcome at the 4^th ^visit for 464 study subjects in a study of 731 chiropractic patients treated for low back pain.

Variables	Subgroups	P-value
***Demographic background and life-style variables***		

Age	*Max. 40 yrs vs. older	0.6

Sex	*Men vs. women	0.5

Type of work	*Sitting vs. walking/standing vs. mixed hard/light work vs. physically hard work	0.4

Smoking	*No never vs. stopped/sometimes vs. daily	0.4

Smokeless tobacco	*No vs. yes	1.0

Treatment reimbursed	*Not or some reimbursed vs. totally reimbursed	**0.001**

***Description of low back pain***		

Leg pain	*No vs. yes	0.8

Pain intensity past 24 hrs	*None/weak vs. moderate vs. severe/unbearable	0.8

Duration of pain at base-line	*1-7 days vs. 8-14 days vs. >14 days	**0.000**

Total duration of pain past year	*Maximum 30 days vs. >30 days	**0.000**

Pain in other parts of spine past year	*No vs. yes max. 30 days vs. yes >30 days	**0.000**

General health	*Very good/good vs. OK vs. rather bad/very bad	**0.03**

Outcome at 4^th ^visit	*Definitely better vs. probably better/unchanged/probably worse/definitely worse/missing data	**0.02**

Pain intensity past 24 hrs at 4^th ^visit	*None/weak vs. moderate vs. severe/unbearable	0.06

***Psychological profile***		

Anxiety	**No vs. borderline vs. yes	0.4

Depression	**No vs. borderline vs. yes	0.3

Cognitive anxiety	***Continuous data tested as mean values with 95%CI	NS

Escape avoidance	***Continuous data tested as mean values with 95% CI	NS

Fearful thoughts	***Continuous data tested as median values with 95% CI	NS

Physiological symptoms And signs of pain	***Continuous data tested as median values with 95% CI	NS

P-values smaller than 0.05 have been written in bold and the corresponding variables have been included in further multivariate analyses

* Chi-square test

**Logistic regression

*** Test for trend, non-significant because confidence intervals overlap

##### ii. Multivariate analysis - 3 months

When all the significant variables from the previous analyses were forced into the model, none emerged statistically significant. However, a stepwise analysis resulted in two significant variables: Duration of pain in the past year (OR 2.1; 95% CI:1.4-3.1) and pain in other parts of spine in the past year (OR 1.6; 95% CI 1.1-2.5). Sensitivity, specificity and area under ROC were all relatively low (60%, 50%, and 62%, respectively), revealing a clinically useless model.

##### iii. Model performance - 3 months

When the number of statistically significant predictive variables from the multivariate analysis was taken into account, a positive gradient was again noted in relation to the outcome. Of those with none of these factors, 68% improved vs. 51% of those with one of the two significant factors but only 41% had a good outcome if they in the past year had more than 30 days of LBP *and *pain in other parts of the spine.

## Discussion

The present study builds on information obtained in similar studies among chiropractors in the Nordic countries. Throughout these studies we have repeatedly found that it is possible, to some degree, to predict outcome but that the final models are fairly weak. That is, the variables tested do not capture what truly predicts outcome. Nevertheless, it was relevant to perform this type of study once more, this time to test the potential importance of a number of psychological variables.

However, it became clear that this type of primary care patients do not present with complicated psychological profiles, nor does the psychological profile predict outcome in those few (less than 10%), who do have serious psychological issues. To be more precise: To add these psychological variables did not improve the predictive model. This confirms the findings from a previous much smaller study of chiropractic patients in the UK [6].

Instead, factors that previously have been shown to affect outcome, such as duration of symptoms, were important both at the 4^th ^visit and after 3 months, whereas general health played a role at the 4^th ^visit and pain in other parts of the spine after 3 months. In other words, factors associated with outcome are chronicity and co-morbidity.

In relation to the 4^th ^visit in the present study, a new variable, fee subsidization entered the scene. Those patients who did not pay for their treatment at all had a worse outcome than those who paid some or all of the fee out of their own pocket. It is not known whether this is because not paying has an effect on the recovery or whether these patients simply represent a different group. In Sweden, free chiropractic treatment is in some instances given to patients who have already paid for a certain number of treatments in the general health care system. It is possible that many of these suffer from chronic illness or that they have one or several co-morbidities that could have an effect on their prognosis also for LBP. This association was however not present at the three months follow-up.

Outcome at the 4^th ^visit was previously shown to predict outcome at three months [3]. In this study, no such finding was evident. This difference can perhaps be explained by the fact that our study consisted both of patients with short-term and long-term/recurrent LBP, whereas the previous study [3] consisted of patients with longer duration or recurrent LBP. The latter group is less likely to have a spontaneous recovery pattern than patients with short-term duration. Therefore, patients with more stable LBP, who will improve with treatment, can probably be found already early in the treatment, whereas a larger proportion of patients with single-event short-lasting LBP will recover and remain improved for a longer time, regardless of treatment.

As in a recent similar study on Finnish chiropractic patients with LBP, there seems to be a clear gradient in relation to the number of positive factors, so that if none of them is present the majority would have a good outcome but only the minority will improve, if more factors are present [5]. This is hardly surprising; that the total burden of unfavourable factors would be more important than single specific variables seems intuitively correct.

Our study has some weaknesses that need to be considered before we can trust the results. Because it is an outcome study without external control group, it is not possible to know if the results would have been the same in patients treated with other methods or even not treated at all. Therefore it is possible that our results merely represent the natural course and that the treatment in this context was superfluous.

The participation rate among chiropractors was rather low, which was anticipated, because of the longish questionnaire. The study was carried out during normal clinic hours on a voluntary basis without any compensation to the participating chiropractors for lost time. Compared to other clinical studies with considerably lower response rates, e.g. as low as 32%, it still appears acceptable [16]. Also, the drop-out analysis did not show any obvious bias that could affect the prediction of outcome, as none of the three variables that differentiated the participants from the drop-outs was related to the significant predictor variables.

The outcome at the 4^th ^visit was recorded by the clinician, to save time and increase the number of returned questionnaires. Obviously, with such a procedure it is possible that the patient provides a pleasing answer (obsequiousness bias). However, in all our studies of this type we have minimized this risk by accepting only "definitely better" as a good outcome. It would also be possible for clinicians to encourage a "good" answer from the patient, or even worse, to falsify the patient's response in order to make the results of the treatment look better. The latter is unlikely, as the chiropractors in our study are used to this type of work and have been well informed many times of the need to find the differences between those who recover and those who do not. Further, at the three months follow-up, outcome was recorded on a questionnaire, which did not result in any illogical results as compared to the clinician-recorded findings, making us assume that the results are fairly accurate.

In LBP research it is customary to use quite complicated outcome questionnaires that measure both pain and disability and various dimensions thereof and their analysis has become a speciality on its own. Such approach is not practical in daily practice because it demands too much time and effort. We have therefore elected to use a clinician-friendly approach of one simple question in relation to outcome with five possible answers (one neutral and two in both directions). This question has provided logical and reproducible results in a number of our studies [4,5,11,12], and can therefore be recommended as a simple and valid method to report outcome in chiropractic patients with LBP. It has the added advantage that this is the sort of question that practitioners normally ask their patients in order to ascertain their clinical development.

This type of study also has some strengths, such as that the patient population and treatment provided are typical of the real clinical situation and that it is a relatively cheap and easy method to collect a sufficiently large amount of data to enable analysis of many variables. Another advantage is that it brings clinicians into the world of research, giving them an understanding of its rigours and sensitises them to the results, once these are published. It is also important to do research into topics that clinicians consider relevant. This study, for example, was initiated because a practicing chiropractor, one of the members of the team, had a special interest in this subject.

## Conclusion

In conclusion, it can be said that it would not appear worthwhile to introduce psychological questionnaires into standard chiropractic practice on patients with LBP. Further, it must be concluded that the predictors chosen did not result in a model particularly helpful to clinicians. It would, however, be relevant for clinicians to keep in mind the negative effect that an accumulation of unfavourable variables can have on outcome. Their presence should make the clinician alert to the need to re-evaluate patients who do not follow the expected recovery pattern to ensure that these patients are not left in long-term treatment programs without a clinical justification.

## Competing interests

The authors declare that they have no competing interests.

## Authors' contributions

AR had the idea of the project and provided the relevant psychological questionnaires. The whole research group planned the project under the supervision of the first author, who also wrote the first draft. IA was responsible for the logistics of the study. Data were collected by all but the first and last authors. CLY and NW laid the strategy for the data analysis. Analysis of the data was made by NW and the whole group participated in the interpretation of data. All participants commented on the manuscript and all authors read and approved of the final manuscript.

## Supplementary Material

Additional file 1 **Brief description of some statistical concepts in the text**. Cross-tabulations, odds ratios, confidence intervals, dose response, multi variate analysis, logistic regression, step-wise logistic regression, sensitivity, specificity and ROC-curve have been briefly explained. Click here for file
