# Supplementary material for: The Nordic Subpopulation Research Programme: prediction of treatment outcome in patients with low back pain treated by chiropractors - does the psychological profile matter?
Source: Chiropr Osteopat. 2009 Dec 30;17:14. doi: 10.1186/1746-1340-17-14 (PMC2807423; doi:10.1186/1746-1340-17-14)
Supplement: Additional file 1 — Brief description of some statistical concepts in the text. Cross-tabulations, odds ratios, confidence intervals, dose response, multi variate analysis, logistic regression, step-wise logistic regression, sensitivity, specificity and ROC-curve have been briefly explained. [file 1746-1340-17-14-S1.DOC]

Additional file 1. Brief description of some statistical concepts in the text

**Cross-tabulations** are used to hold one variable e.g. gender (man/woman) up against another variable e.g. outcome (definitely better/not definitely better).

The results can be reported as percentages (e.g. 30% of men were definitely better and 45% of the women) but it is often reported as an **odds ratio** (OR), which is the ratio of odds between two groups. It is often used to describe the strength of an association, such as the association between a clinical finding and treatment outcome. In our study, a value greater than 1 indicates a positive association, i.e. that those with a positive clinical finding are more likely than men to have a good outcome than those with a negative finding, and the higher this figure the stronger the association. ORs between 1 and 2 are commonly found in studies of back pain but such values are not particularly impressive. ORs above 2 are more interesting whereas an OR below 1 indicates a negative association.

Commonly this association is obtained using a chi-square test, which has the advantage that it can be done by hand. However, more sophisticated methods are also available, such as a logistic regression, which requires the use of a specific soft-ware program.

When the data are reported as a continuous variable (such is often the case with biological measurements) and not as categories (man/woman; yes/no), other methods must be used to detect differences between values. We used test for trend, in our study, to see if the psychological score played a role in relation to outcome. A test for trend is used to test if there is consistency of the results in the same direction.

Because this study does not include all chiropractic patients with low back pain in the world, our results are only considered to be estimates of the true answer. In order to deal with the uncertainty as to whether these results are generalizable or not, **confidence intervals** (CI) are used. A 95% CI is an interval at both sides of the estimated OR that indicates that if the same study was conducted 100 times taking patients from the same underlying study population and with the same sample size, 95% of these studies would produce an estimated OR within the borders of the low and high limits of the CIs. If the lower limit is greater than 1, then the OR is considered to be “significant”. For example, if the OR is surrounded by a CI going from 1.5 to 2.5, the OR is said to be significant. In other words, it is likely that there is in fact a positive association. If, the OR instead is surrounded by a CI going from 0.8 to 2.8, it is not likely to be significant, as the lower CI lies below 1. In addition, the width of the CI provides an idea of the precision of the estimate; the shorter the distance between the two endpoints the more precise the estimate.

A **positive gradient (or a dose-response)** is said to exist when increasing exposures increase the risk of disease. For example, non-smokers would not be very likely to have chronic bronchitis, whereas it would be more common in those who smoke a little, and those who smoke the most will be most likely to have this disease. Also the number of risk factors can exhibit a dose-response, so that people with no risk factors are least likely to have a disease but the larger the number of risk factors the higher the percentage of people with the disease. This is what we found in our study.

Even if there is an association between two variables it is not certain that the one causes the other. It is not even sure that this association is real. In order to find out if this is the case, it would often be necessary to control for the effect of other variables, in a **multivariate analysis** (MVA). One method often used is called “**logistic regression**”. In a logistic regression, combinations of the variables are tested against the outcome, to see which ones – if any – are really linked with the outcome variable. It requires the use of an outcome variable with only two possible answers (e.g. definitely better yes/no as in our study), and the estimates will be reported as ORs, surrounded by CIs, which tell us whether the estimates are significantly positive or not and the precision of the estimates (see above). However, we still do not know if there is a cause or not. The question of causality is more complex than so.

Another type of MVA is called “**stepwise logistic regression**”. With this method, the original bivariate test is complemented by the gradual inclusion and exclusion of all the other variables, until the “best” results emerge. In this way, irrelevant variables are removed and the variables that emerge from a MVA are usually independently associated with the outcome variable, i.e. that the association is real.

**Sensitivity**: The usual definition is “the percentage of sick people who are identified as having the condition”. In this article, it signifies the percentage of people with a certain profile who could be identified as becoming “definitely better”.

**Specificity**: The usual definition is “the percentage of well people who are identified as not having the condition”. In this article it signifies the percentage of people who do not have a certain profile who could be identified as not becoming “definitely better”.

An additional method to describe how well a variable (such as a clinical test) or a number of variables (such as several clinical tests together) can explain an outcome, is to test statistically how big an area they occupy under the so-called receiver operating characteristics (**ROC**) curve. A ROC value of 50% indicates chance findings, whereas the minimal value of 70% arbitrarily is considered as acceptable, and 100% is considered perfect.
